# Effect of pigtail catheter application on obstetric outcomes in in vitro fertilization/intracytoplasmic sperm injection pregnancies following hyperstimulation syndrome

**DOI:** 10.4274/tjod.33340

**Published:** 2017-06-15

**Authors:** Pınar Çağlar Aytaç, Hakan Kalaycı, Selçuk Yetkinel, Didem Alkaş, Seda Yüksel Şimşek, Bülent Haydardedeoğlu, Esra Bulgan Kılıçdağ

**Affiliations:** 1 Başkent University Faculty of Medicine, Department of Obstetrics and Gynecology, Division of Reproductive Endocrinology and IVF, Adana, Turkey; 2 Başkent University Faculty of Medicine, Department of Obstetrics and Gynecology, Division of Perinatology, Adana, Turkey; 3 Ağrı State Hospital, Clinic of Obstetrics and Gynecology, Ağrı, Turkey; 4 Kadirli State Hospital, Clinic of Obstetrics and Gynecology, Osmaniye, Turkey

**Keywords:** Ovarian hyperstimulation syndrome, ascites, pigtail catheter, in vitro fertilization

## Abstract

**Objective:**

To evaluate the effects of percutaneous pigtail catheter drainage on the outcomes of intracytoplasmic sperm injection (ICSI) pregnancies following moderate or severe ovarian hyperstimulation syndrome (OHSS).

**Materials and Methods:**

This retrospective study included 189 patients hospitalized for OHSS following ICSI treatment in a tertiary in vitro fertilization unit between 2006 and 2014. Pigtail catheters were applied in 63 patients; the other 126 patients did not need that treatment. The obstetric reports of 173 patients could be accessed and were examined to investigate the pregnancy outcomes of those with and without catheters.

**Results:**

No complications such as infection or vascular or intra-abdominal organ trauma were observed related to the pigtail application. There were no differences in abortus, preterm labor, gestational diabetes mellitus, and preeclampsia ratio between the pigtail and control groups (p>0.05). The rate of readmission to hospital for OHSS was lower in the pigtail group than in the control group although not statistically significant (p=0.08).

**Conclusion:**

Pigtail application is a safe and effective method for draining ascites in patients with OHSS after ICSI treatment. The use of pigtail catheters had no adverse effects on the perinatal outcomes of patients hospitalized with OHSS who became pregnant after ICSI treatment. In addition, the percutaneous drainage of ascites via a pigtail catheter helped prevent the readmission of patients with moderate or severe OHSS.

## PRECIS:

Pigtail application is a safe and effective in draining ascites in patients with ovarian hyperstimulation syndrome after intracytoplasmic sperm injection treatment and had no adverse effects on perinatal outcomes.

## INTRODUCTION

Ovarian hyperstimulation syndrome (OHSS) is a serious and sometimes life-threatening complication of in vitro fertilization (IVF) treatment. Although various preventive measures are taken, the incidence of OHSS is still around 2%^(1)^, and management plays an important role in this potentially lethal complication. There are no OHSS-free clinics, although there are preventive measures such as freeze-all protocols and lower ovarian stimulation IVF protocols.

Mild-to-severe forms of clinical findings such as leukocytosis, hemoconcentration, hypovolemia, edema, ascites, hydrothorax, pericardial effusion, thromboembolic events, oligo or anuria, acute respiratory distress syndrome, and multiple organ failure can be seen due to extravasation of intravascular protein-rich fluid to the third spaces. Ascites form as a result of the underlying pathophysiology and aggravate the course of the disease. In the management of OHSS, it is necessary to eliminate the ascites to alleviate symptoms, which include abdominal discomfort and dyspnea. Abdominal paracentesis may need to be performed several times, and it is usually done blindly. An easier and safer method of draining ascites is to use a pigtail catheter, which is inserted easily into the abdomen percutaneously under local anesthesia and guided using ultrasonography^(2)^. Pigtail catheters have proven to be safe for draining ascites fluid. However, it is not clear whether this invasive procedure creates any procedure-related risk for pregnant patients such as bleeding, infection, trauma to neighbouring intraabdominal organs or preterm delivery.

To the best of our knowledge, there are no data comparing the obstetric outcomes of patients with pigtail catheters and controls. In our study, therefore, we evaluated the obstetric outcomes of patients who experienced an IVF/intracytoplasmic sperm injection (IVF/ICSI) pregnancy following OHSS, and compared patients with and without pigtail catheters.

## MATERIALS AND METHODS

### Patient population

Between May 2006 and September 2014, a total of 9289 consecutive IVF/ICSI cycles were performed at our University Hospital, Department of Obstetrics and Gynecology, Division of Reproductive Endocrinology and IVF Unit. One hundred and eighty-nine number of patients who were hospitalized due to moderate or severe OHSS with ascites were included in the study. Those whose obstetric data could not be obtained were excluded. We could be able to access the obstetric outcomes of 173 number of patients treated with or without pigtail catheters in two separate groups. Abortus ratios, preterm labor ratios, preterm premature rupture of membrane (PPROM) ratios, and other pregnancy outcomes such as preeclampsia, intrauterine growth retardation (IUGR), gestational diabetes mellitus (GDM), and birth weights and birth weeks of gestations were analyzed between the two groups. The study was approved by the ethics committee of the university. This is a retrospective study; therefore, informed consent was not required from the patients.

### Ovarian hyperstimulation syndrome treatment and follow-up

The classification of OHSS was defined according to the criteria of Golan et al.^(3)^ and Navot et al.^(4)^. Moderate OHSS was defined as abdominal distention and discomfort, nausea, vomiting, diarrhea, enlarged ovaries 5-12 cm in diameter, and presence of ascites. Severe OHSS was defined as massive ascites, hydrothorax, breathing difficulties, hematocrit >45%, leukocytes >15.000, creatinine clearance ≤50 mL/min, oliguria, and liver dysfunction.

In our clinic, hospitalization is recommended for patients with moderate and severe OHSS to facilitate close monitoring. During follow-up, patients with OHSS are treated with bed rest, and the following measurements are taken every day: body weight, waist circumference, vital signs every four hours, urine output, and intravenous or oral fluid intake. Total daily fluid intake is limited to 3000 mL/day. If the patient cannot tolerate oral fluid intake, parenteral replacement is administered. Hydroxyethyl starch (HES) solution (6% HES 130/0.4 in 0.9% sodium chloride injection; Voluven^®^, Germany) is added to the parenteral solution to inhibit the shifting of intravenous fluid to the extracellular plain. If abdominal distention and discomfort are too high and oliguria is present, a pigtail catheter (8-0 French Flexima Drainage Catheter^®^; Boston Scientific, USA) is applied. During follow-up, if the ascites does not regress, a human albumin (human albumin 20%, 100 mL; Octapharma, Austria) infusion is given to patients who have decreased serum albumin levels. To prevent deep venous thrombosis, it is suggested that patients wear venous support stockings, and low-molecular-weight heparin (5000 units per day) is ordered. When morning hematocrit levels are ≤38% or the patients’ symptoms are alleviated, discharge from the hospital is recommended.

The following patient data were recorded: age; infertility duration; IVF indication; IVF protocol; total gonadotropin doses used in IVF; estradiol level on human chorionic gonadotropin (hCG) day; number of follicles collected on oocyte pickup (OPU) day; total HES or albumin solution infused during treatment; hematocrit, leukocyte, platelet, and electrolyte counts on the days of admission to and discharge from hospital; length of stay in hospital; readmission to hospital, if necessary; clinical pregnancy and live birth rate; singleton, twin, or triplet pregnancy; and infant birth weight.

During the obstetric follow-up, the following tests were administered to diagnosis GDM. First, a 50-g oral glucose challenge test was performed between 24-28 weeks of gestation; if the first-hour glucose level was >140 mg/dL, a 100-g oral glucose tolerance test (OGTT) was performed. In the case of familial history of diabetes mellitus or history of GDM during previous pregnancy, OGTT is suggested just after the first visit. Fasting, first-, second-, and third-hour glucose values greater than 95, 180, 155, and 140 mg/dL, respectively, were diagnosed as GDM. Preeclampsia was defined as blood pressure ≥140/90 mmHg measured at two different times and ≥300 mg proteinuria in 24-hour collected urine. Preterm labor was defined as the beginning of uterine contractions prior to 37 weeks’ gestation. PPROM was defined as rupture of the amniotic membrane prior to 37 weeks’ gestation. Intrauterine growth restriction was defined as weight below the tenth percentile for gestational age, which was measured sonographically. Miscarriage was categorized as either early (before 12 weeks’ gestation), or late (after the 12^th^ week). A chemical abortus was diagnosed in the presence of elevated hCG level in the patient’s serum without demonstrating a gestational sac in the transvaginal sonographic examination.

### Statistical Analysis

In the statistical analyses, demographic data of the patients are given as observational values, percentage rates, mean values, and standard deviation values. The chi-square test was used in the correlation analysis of categorical data while comparing the groups, and the Mann-Whitney U test was used in the paired comparison of continuous data, which generally did not comply with normal distribution. In addition, observation numbers obtained on the basis of catheter status were compared by applying the method of comparison of two rates. The data were analyzed using SPSS for Windows version 18.0 (SPSS, Inc., USA).

## RESULTS

Between 2006 and 2014, 9289 patients underwent IVF/ICSI procedures at the University Hospital, Department of Obstetrics and Gynecology, Division of Reproductive Endocrinology and IVF Unit. Of those patients, 189 (2%) were hospitalized due to severe or moderate OHSS.

Of the 189 patients hospitalized due to OHSS, 33.3% (n=63) required a pigtail catheter and 66.6% (n=126) did not need catheterization for abdominal ascites drainage. We found no differences between the two groups in terms of age; total gonadotropin dose given during IVF treatment; estradiol blood level on the day of hCG; oocyte number after OPU; duration of infertility; or hemoglobin, hematocrit, and platelet levels on the first and last days of hospitalization. With the exception of the last day, the catheterized patients had higher platelet levels. In addition, the length of hospitalization was longer (p<0.01) and the HES and human albumin solution levels used were higher (p<0.01) in the catheterized group (Table 1). The duration of catheterization was 1-9 days in the catheterized group (mean: 2.9±1.7 days). After being discharged from hospital, 27 (14.3%) patients needed to be readmitted to hospital due to OHSS. In the catheterized group, the readmission ratio was 7.9%, whereas in the non-catheterized group, the readmission ratio was 17.5% (p=0.08) (Table 1).

The ratios of singleton, twin, and triplet pregnancies in the two groups were statistically similar, but the twin pregnancy ratio was higher in the catheterized group (Table 2). The miscarriage ratios, whether early miscarriage or chemical abortus, were statistically similar between the two groups (p>0.05) (Table 2).

The clinical pregnancy ratio of the catheterized group was higher than that of the non-catheterized group (p<0.01). In addition, the catheterized group had a statistically higher live birth ratio (p=0.04) (Table 2). The early OHSS ratio was higher in the non-catheterized group, and the late OHSS ratio was higher in the catheterized group (p=0.01).

The perinatal outcomes of the two groups, such as preterm labor, GDM, preeclampsia, PPROM, IUGR, and miscarriage ratios, were similar (Table 2).

The birth weights and gestational ages of the twin and singleton pregnancies were compared. The mean birth weight and gestational age at delivery of twins in the catheterized group were 2085 g and 34.6 weeks, respectively; in the non-catheterized group, these values were 2200 g and 35 weeks, respectively (p=0.65). The mean birth weight and gestational age at delivery of singleton babies in the catheterized group were 3251 g and 37.6 weeks, respectively; in the non-catheterized group, these values were 2728 g and 36.6 weeks, respectively (p=0.08) (Table 2).

Regression analyses of live birth rates of the two groups were performed, the catheter presence and other factors did not affect the live birth rates of the two groups (p>0.05) (Table 3).

## DISCUSSION

In our study, we compared the complications and obstetric outcomes of patients who had pigtail catheters applied for ascites drainage with those of patients who did not require catheterization. Pigtail catheter application, although conducted under the guidance of ultrasonography, is still an invasive method. During the procedure, the abdominal wall is punctured, which can cause peritoneal irritation or secondary infections related to the process itself, possibly resulting in fetal or maternal complications. We encountered no complications related to pigtail catheter application, such as infection, or bowel or other intra-abdominal organ trauma. We know that the inflammatory process can cause the release of cytokines and chemokines that can precipitate preterm labor^(5)^.

Obstetric outcomes such as preterm labor, early and late miscarriages, preeclampsia, GDM, PPROM, IUGR, and fetal anomaly were compared between the two groups, and we found no differences. The analysis of miscarriage rates, including the subgroups early, late, and chemical miscarriage, also revealed similarities between the two groups (Table 2). Overall, the miscarriage ratio of the patients who had ascites drainage with pigtail catheter was 26.2%. The miscarriage ratio which was 13.2% in the control group was lower than the catheter group, but the difference was not statistically significant. The higher ratios of miscarriage could be explained by higher rates of OHSS ratios in the catheterized group than in the control group. Most of the patients who were hyperstimulated had polycystic ovaries and the miscarriage rate after IVF treatment is higher in patients with polycystic ovary syndrome (PCOS)^(6,7)^. In our groups, the numbers of patients with PCOS were similar; the higher ratios of miscarriage was not higher than the miscarriages rates of other studies such as Chen et al.^(8)^ (26.6%), Serdyńska-Szuster et al.^(9)^ (26.9%), and Raziel et al.^(1)^ (38%).

Multiple pregnancy is a risk factor for preterm labor; however, although not statistically significant (p=0.06), the rate of twin pregnancy was higher in the pigtail group. The preterm labor ratios did not differ between the groups (p=0.28). In the literature, the preterm labor rate in patients with OHSS after IVF treatment varies from 1% to 28%^(10,11,12)^. In the study by Haas et al.^(13)^, pregnancy outcomes in patients with severe OHSS following ascetic drainage were evaluated, and their preterm labor risk was found as 12.5%. In our study group, the preterm labor risk was 18.5%, and that of the control group was 19% (p>0.05). However, the Haas et al.^(13)^ study included a small case sample (n=16), they had no control group, and most importantly, the pregnancies were all singletons. Even though twins and triplets were included in our study, the overall preterm labor rate was not much higher than that of the Haas et al.^(13)^ study.

In our study, there were no differences between the groups in terms of birth weight or gestation age at delivery. The results regarding pregnancy outcomes after OHSS in IVF treatment revealed no differences between the groups and they were better than those in the review of Raziel et al.^(14)^.

The rates of risk factors for developing OHSS, such as age, diagnosis of PCOS, total dose of gonadotropins during controlled ovarian hyperstimulation, estradiol level on the day of hCG, and oocyte numbers, were similar between the two groups. However, the catheterized group had a longer duration of hospitalization, they required greater amounts of HES and human albumin solution, and their platelet levels were higher on the day of discharge from hospital. These results were not entirely unexpected, because the ascites in the catheterized group were more severe, thus requiring the pigtail catheter. Although the pigtail group had a more severe form of OHSS, the mean duration of hospitalization, i.e. the recovery time from OHSS was 7.7 days; however, in the study of Nouri et al.^(15)^, the recovery time from OHSS was 9 days. Even if the pigtail group had a longer hospitalization time than the control group, it was still lower than the cases in the study of Nouri et al.^(15)^. Application of a pigtail catheter may not increase the length of hospitalization, but the severity of ascites might slow the recovery process.

Then clinical pregnancy ratio, live birth ratio per cycle, and late OHSS ratios were higher in the pigtail group than in the control group (p<0.05) (Table 2). When we analyzed the regression analysis of factors that effected the live birth rate, we found no significance between them (Table 3). The higher late OHSS ratios in the pigtail group can be reasonably explained by the higher pregnancy rate. In addition, it is known that the pregnancy rate is higher among patients undergoing IVF/ICSI who had OHSS^(1)^. Although not statistically significant, the catheterized group had a lower rate of hospital readmission than the non-catheterized group, despite the fact that the catheters were used in patients who were more clinically severe. This finding suggests that in managing OHSS, use of the pigtail catheter has an advantage in that it can help decrease hospital readmission rates.

### Study Limitations

The major limitation of our study is its retrospective nature. Pigtail application is an invasive procedure, as such we did not have the chance to randomize the patients who had severe OHSS. Abdominal paracentesis may be an alternative to the pigtail catheter, but abdominal paracentesis needs to be applied blindly everyday through the abdominal wall. Pigtail application for drainage of ascites is made only once, thus the single application may be an advantage over repetitive abdominal paracentesis, which has not been practiced in our clinic for many years.

## CONCLUSION

The application of pigtail catheters did not decrease the pregnancy ratio or live birth ratio in patients with OHSS. In addition, the hospital readmission rate was lower. The obstetric outcomes were similar between both groups. Thus, percutaneous pigtail catheters can be used in the treatment of ascites in patients with OHSS without raising concerns regarding pregnancy outcomes.

## Figures and Tables

**Table 1:**
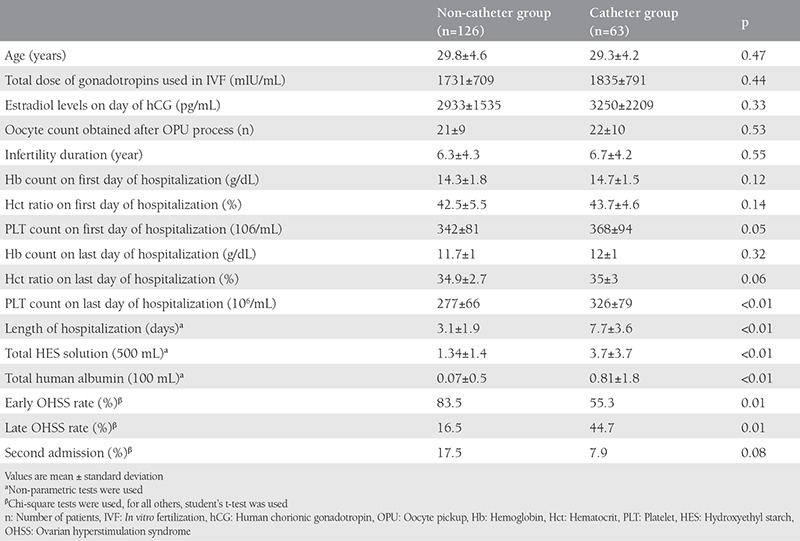
Characteristics of the two groups

**Table 2:**
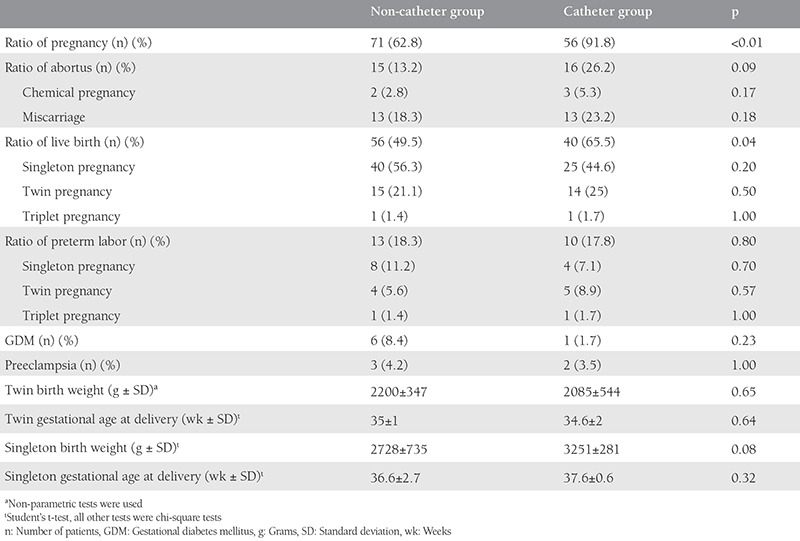
Obstetric outcomes of the two groups

**Table 3:**
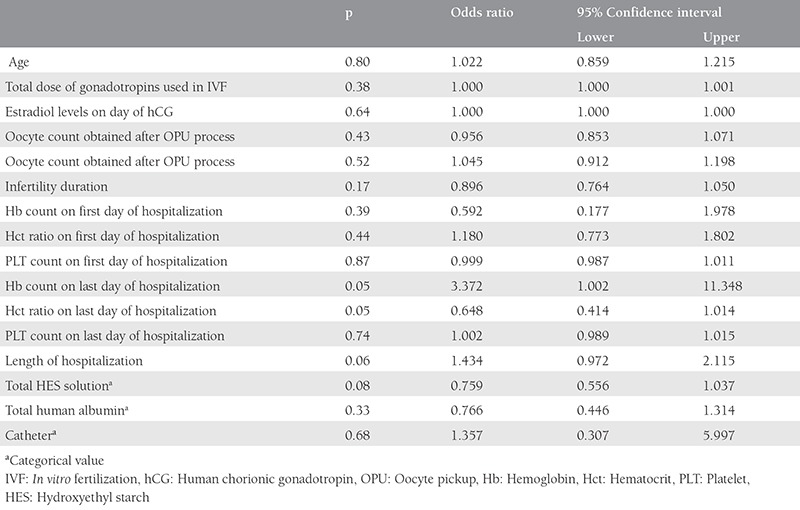
Logistic regression of factors effecting live birth rates

## References

[1] Raziel A, Friedler S, Schachter M, Strassburger D, Mordechai E, Ron-El R (2002). Increased early pregnancy loss in IVF patients with severe ovarian hyperstimulation syndrome. Hum Reprod.

[2] Abuzeid MI, Nassar Z, Massaad Z, Weiss M, Ashraf M, Fakih M (2003). Pigtail catheter for the treatment of ascites associated with ovarian hyperstimulation syndrome. Hum Reprod.

[3] Golan A, Ron-el R, Herman A, Soffer Y (1989). Ovarian hyperstimulation syndrome: an update review. Obstet Gynecol Surv.

[4] Navot D, Bergh P, Laufer N (1992). Ovarian hyperstimulation syndrome in novel reproductive technologies: prevention and treatment. Fertil Steril.

[5] Tency I (2014). Inflammatory response in maternal serum during preterm labour. Facts Views Vis Obgyn.

[6] Liu L, Tong X, Jiang L, Li T, Zhou F, Zhang S (2014). A comparison of the miscarriage rate between women with and without polycystic ovarian syndrome undergoing IVF treatment. Eur J Obstet Gynecol Reprod Biol.

[7] Wang XX, Luan CX, Zhang W, Hu SM (2012). [Pregnancy outcomes of in vitro fertilization and embryo transfer in infertile women with polycystic ovarian syndrome]. Zhonghua Fu Chan Ke Za Zhi.

[8] Chen C, Wu M, Chao K, Chen S, Ho H, Yang Y (1997). Serum estradiol level and oocyte number in predicting severe ovarian hyperstimulation syndrome. J Formos Med Assoc.

[9] Serdynska-Szuster M, Jedrzejczak P, Ozegowska K, Korman M, Pawelczyk L (2012). [Perinatal outcome among women undergoing in vitro fertilization procedures complicated by ovarian hyperstimulation syndrome]. Ginekol Pol.

[10] Abramov Y, Elchalal U, Schenker JG (1998). Obstetric outcome of in vitro fertilized pregnancies complicated by severe ovarian hyperstimulation syndrome: a multicenter study. Fertil Steril.

[11] Courbiere B, Oborski V, Braunstein D, Desparoir A, Noizet A, Gamerre M (2011). Obstetric outcome of women with in vitro fertilization pregnancies hospitalized for ovarian hyperstimulation syndrome: a case-control study. Fertil Steril.

[12] Wiser A, Levron J, Kreizer D, Achiron R, Shrim A, Schiff E, et al (2005). Outcome of pregnancies complicated by severe ovarian hyperstimulation syndrome (OHSS): a follow-up beyond the second trimester. Hum Reprod.

[13] Haas J, Yinon Y, Meridor K, Orvieto R (2014). Pregnancy outcome in severe OHSS patients following ascitic/plerural fluid drainage. J Ovarian Res.

[14] Raziel A, Schachter M, Friedler S, Ron-El R (2009). Outcome of IVF pregnancies following severe OHSS. Reprod Biomed Online.

[15] Nouri K, Tempfer CB, Lenart C, Windischbauer L, Walch K, Promberger R, et al (2014). Predictive factors for recovery time in patients suffering from severe OHSS. Reprod Biol Endocrinol.

